# FiO_2_/PEEP index: a simple tool for opitimizing ventilator settings

**DOI:** 10.1186/cc12028

**Published:** 2013-03-19

**Authors:** D Trasy, M Nemeth, K Kiss, Z Till, Z Molnar

**Affiliations:** 1 University of Szeged, Hungary

## Introduction

During mechanical ventilation, oxygenation can be influenced by adjusting FiO_2 _and positive end-expiratory pressure (PEEP). There have been recommendations for how the FiO_2 _and PEEP should be set [1]. However, in a recent audit we found that the compliance of doctors of these recommendations is very low [2]. Therefore we invented a simple parameter called the FiO_2_/PEEP index (FPi) of which the physiologic value is ≤7 (that is, FiO_2 _= 21%/PEEP = 3 cmH_2_O), which corresponds to the ARDSNet trial's minimum FiO_2_/PEEP settings: 35%/5 cmH_2_O [2]. The aim of this case-control study was to investigate the impact of an FPi ≤7 targeted protocol on clinical practice.

## Methods

A prospective observational study in 2010 and 2012. Every mechanically ventilated patient was recruited. Demographics, outcome data and Lung Injury Score (LIS) were recorded after admission. Respiratory settings, oxygenation and ventilation parameters were recorded after mechanical ventilation was commenced and the first arterial blood gas sample was taken (T0). Measurements were repeated in 24 hours (T1). Data are presented as mean ± SD, paired-sample and independent-sample *t *test and chi-square tests were used for statistics.

## Results

In 2010, 75 patients, and in 2012, 130 patients were included. There was no difference in demographics, disease severity, but LIS was higher in 2012: 1.34 ± 1.13 versus 0.84 ± 0.98, *P *= 0.001. There was no significant difference in FPi between the two groups at T0: 10.91 ± 4.25 versus 10.26 ± 5.01 (2012 vs. 2010, respectively). At T24 the FPi was significantly lower in 2012 as compared with 2010: 7.28 ± 2.58 versus 8.17 ± 3.3, *P *= 0.001; which was due to the higher PEEP applied: 7.08 ± 2.87 versus 6.63 ± 2.91, *P *= 0.014. Although in 2012 significantly more patients, 112 (86%), were ventilated with FiO_2 _≥50% at T0 as compared with 2010, 44 (58%) (*P *= 0.001), by T24 significantly less patients received FiO_2 _≥50%, 46 (35%) vs. 34 (45%) (*P *= 0.011). There was no significant difference between the two groups regarding FiO_2_, PaO_2 _and PaCO_2 _at T0 and T24.

## Conclusion

Implementing an FPi ≤7-based algorithm significantly reduced the FiO_2 _and increased the PEEP applied in mechanically ventilated within the first 24 hours. Whether this has any impact on earlier weaning due to reaching the weaning criteria of FiO_2 _sooner, and as a result shortening the duration of mechanical ventilation, has to be investigated in the future.

## References

[B1] ARDSNt N Engl J Med 2000 17 1301

[B2] Kiss K Intensive Care Med 2011 17 Suppl 2 S195

